# Chronic maxillary sinusitis of dental origin and oroantral fistula: The results of combined surgical approach in an Italian university hospital

**DOI:** 10.17305/bjbms.2020.4748

**Published:** 2020-11

**Authors:** Massimo Galli, Giulia De Soccio, Fabrizio Cialente, Francesca Candelori, Francesca Romana Federici, Massimo Ralli, Marco de Vincentiis, Antonio Minni

**Affiliations:** 1 Department of Oral and Maxillofacial Science, University Sapienza of Rome, Rome, Italy; 2 Department of Sense Organs, University Sapienza of Rome, Rome, Italy

**Keywords:** Chronic maxillary sinusitis of dental origin, oroantral fistula, odontogenic sinusitis, maxillary sinus disease, endoscopic sinus surgery, ostiomeatal complex, Lund-Mackay grading, Rehrmann flap

## Abstract

Unilateral chronic maxillary sinusitis is a possible complication of odontogenic disease or dental treatment and is mainly due to the development of an oroantral fistula (OAF). The management of chronic maxillary sinusitis of dental origin (CMSDO) requires a combined treatment via endoscopic sinus surgery (ESS) and intraoral surgical treatment of the odontogenic source. The aim of this study is to present the results of our university hospital unit in the treatment and follow-up of a case series of 34 patients treated with a combined surgical approach for CMSDO due to OAF. All patients were treated with ESS combined with an intraoral approach. No intraoperative or immediate postoperative complications were observed; nasal synechia was found in 3 patients (8.82%). The overall success rate after the primary intervention was 94.12%; recurrence was observed in 2 cases (5.88%), both were suffering from diabetes mellitus and were tobacco smokers. Our results confirm that simultaneous surgery with a combination of an intraoral and endoscopic approach can be considered the best strategy for the long-term restoration of normal sinonasal homeostasis in selected patients with chronic odontogenic sinusitis and OAF, guaranteeing an effective treatment with minimal complications in the short and long term.

## INTRODUCTION

Chronic maxillary sinusitis of dental origin (CMSDO) represents a frequent condition that accounts for 10% to 12% cases of maxillary sinusitis [1-3].

Oroantral fistula (OAF), an unnatural communication between the oral cavity and maxillary sinus with epithelialization in the fistula tract, is among the most common causes of CMSDO, accounting for approximately 60% of odontogenic sinusitis cases [4,5]. OAF mainly follows the extraction of upper molar and premolar teeth; other causes include periapical abscess, periodontal disease, placement of dental implants, maxillary cystic lesions, or foreign bodies such as endodontic materials and dental fillings [6].

CMSDO must be suspected in patients with unilateral symptoms that do not respond to standard medical therapy, and in those who have a history of dental surgical treatment or dental pain [4,7-9].

**FIGURE 1:**
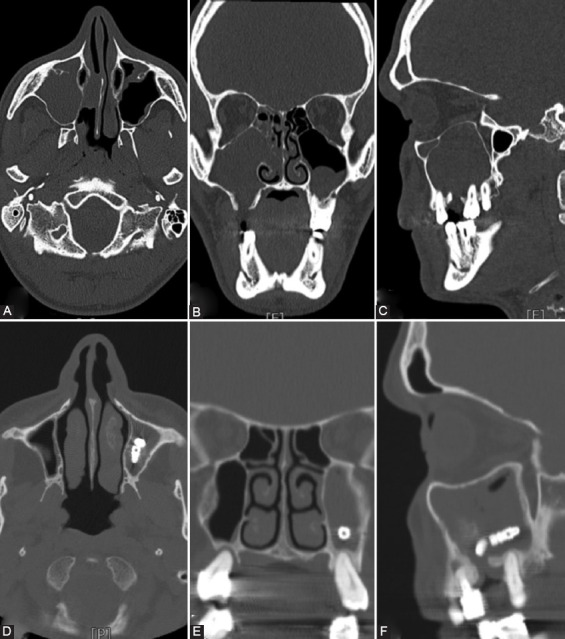
Upper panel: computed tomography scan in the axial (A), coronal (B), and sagittal (C) planes of a patient with right maxillary sinusitis and a large oroantral fistula. Lower panel: computed tomography scan in the axial (D), coronal (E), and sagittal (F) planes of a patient with a wide floor defect of the left maxillary sinus associated with implant displacement and complete sinus obliteration.

High-resolution computed tomography (CT) scans and cone-beam volumetric CT (CBCT) can support in the identification of the dental disease and odontogenic sinusitis (Figure 1) [10,11]. The management of CMSDO requires sinusitis treatment via endoscopic sinus surgery (ESS) as well as intraoral surgical treatment of the odontogenic source through a combined approach [12-17].

The aim of this study is to present the results of our university hospital in the treatment and follow-up of a case series of 34 patients treated with a combined surgical approach for CMSDO due to OAF.

## MATERIALS AND METHODS

Between January 2010 and December 2019, 34 patients with chronic maxillary sinusitis and related chronic OAF were treated by ESS and intraoral OAF closure at the Policlinico Umberto I of Rome. The inclusion criteria were age >18 years, presence of chronic OAF following tooth extraction, and clinical and radiological evidence of ipsilateral chronic maxillary sinusitis.

All patients underwent preoperative otolaryngology and dental clinical evaluation; orthopantomography, axial and coronal contiguous 1 mm CT or CBCT scans of paranasal sinuses, and nasal endoscopy were performed to identify the location and extent of the disease.

The following information was collected during the initial visit for each patient: gender and age, comorbidities, and history of smoking.

### Surgical intervention

Surgery was performed under general anesthesia by the same surgeons. Local infiltration of the middle turbinate and uncinate process with a local anesthetic solution containing epinephrine was performed to minimize bleeding. Medial traction of the middle turbinate and retrograde resection of the posteroinferior part of the uncinate process was performed using a rigid 30° 4 mm endoscope (Karl Storz, Tuttlingen, Germany). The uncinate process cut edges were trimmed with a microdebrider to identify the natural maxillary sinus ostium. The ostium was enlarged in a posteroinferior direction to allow a clear visualization of the natural sinus and its drainage after healing. Foreign bodies were removed through the enlarged maxillary ostium using a 45° and 70° 4 mm endoscope and a curved suction tip. Polyps – if present – were removed, while edematous and hyperemic mucosa was preserved. After ESS, the oral surgery team performed the necessary tooth extractions and removal of pre-existing prostheses or dental implants. Subsequently, the OAF was excised, allowing correct evaluation of the size of the bony defect and clear access to the alveolar recess of the maxillary sinus. A buccal advancement flap procedure according to the Rehrmann technique was performed to reach a complete closure of the fistulous defect.

The following information was collected during the first postoperative visit for each patient: surgical technique and intra and postoperative complications, postoperative use of analgesics and antibiotics, and duration of hospitalization.

### Ethical statement

The study was approved by the local Ethics Committee of our University Department and was performed in accordance with the Helsinki declaration and its amendments. Informed consent was obtained from all the participants.

### Statistical analysis

Descriptive analysis was used to define the main clinical and demographic characteristics of the patients. Data were expressed as means and percentages. GraphPad Prism version 8.3.1 for Windows (GraphPad Software, La Jolla California USA) was used to perform statistical analysis.

## RESULTS

This study included 34 patients with unilateral OAF situated in the alveolar region. Ten patients were women (29.42%) and 24 were men (70.58%). Patients were aged between 20 and 78 years (mean: 52.63 years).

All patients had a diagnosis of unilateral CMSDO with OAF. In 17 patients (50%), anterior ethmoid sinusitis was also present; 30 patients (88.23%) had an obstruction of the ostiomeatal complex. At the time of surgery, unilateral purulent rhinorrhea was the most common presenting sign in 20 patients (58.82%), followed by unilateral nasal obstruction in 17 patients (50%), postnasal drip in 9 patients (26.47%), hyposmia in 8 patients (23.5%), headache in 7 patients (20.58%), unpleasant smell sensation in 6 patients (17.64%), facial pain in 5 patients (14.7%), and swollen cheek in 5 patients (14.7%) (Table 1).

**TABLE 1:**
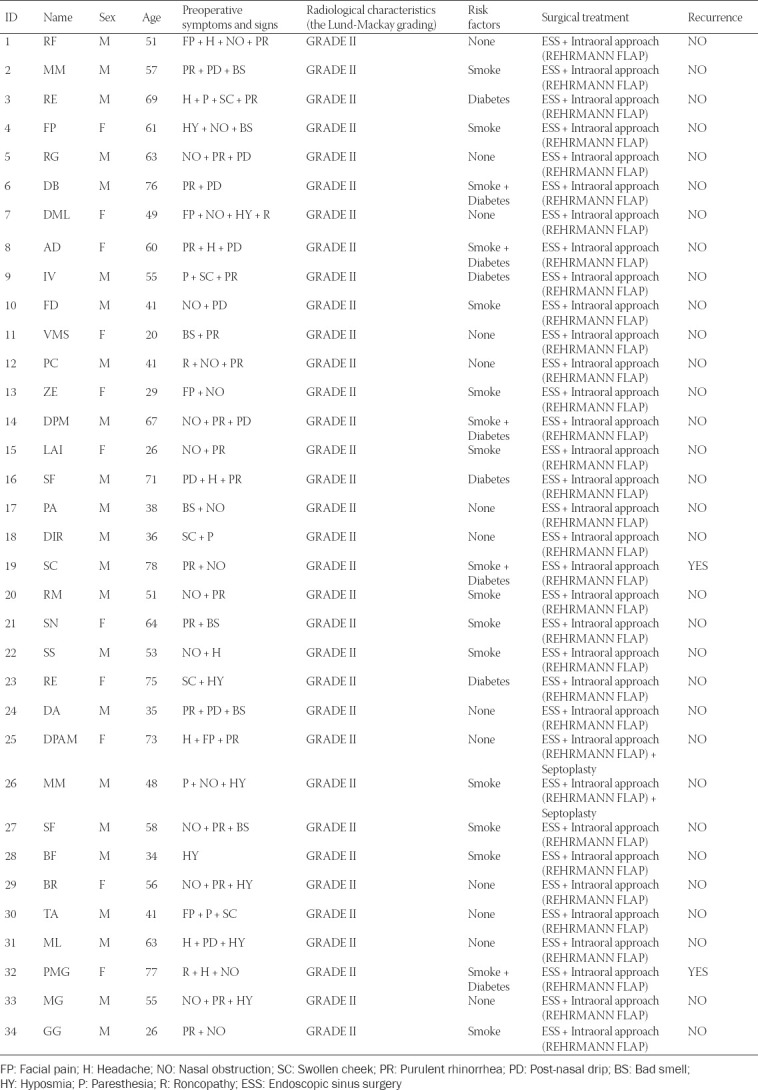
Demographic and clinical data of patients included in the study

**FIGURE 2:**
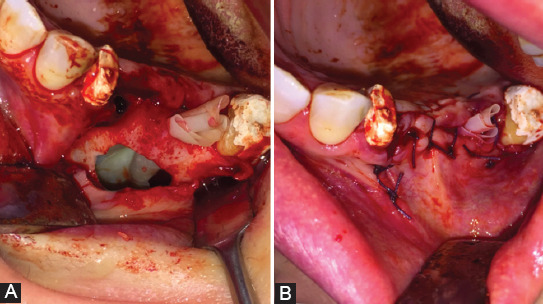
(A) Intraoral surgical approach. A large bony defect was found after the extraction of two molars. (B) The Rehrmann flap was closed with a free-tension flap and eversion to avoid wound dehiscence.

**FIGURE 3:**
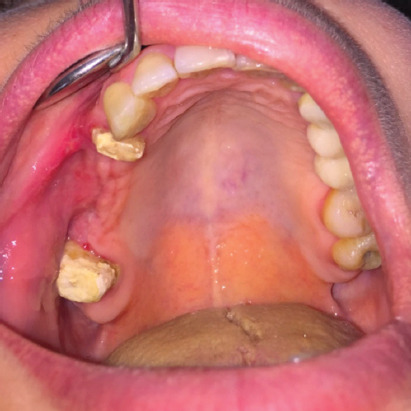
Two months after surgery, complete wound healing was observed.

All patients were classified as grade II according to the Lund-Mackay grading and were treated with ESS combined with an intraoral approach (Figure 2). Postoperative hospitalization was 1 or 2 days (average 1.3 days). Patients were instructed to avoid using straws, smoking, and all the activities that could cause pressure changes between the nasal and oral cavities for at least 1 month. Sutures were removed 7 days after surgery and postoperative follow-up visits were made every 6 months for 1 year to exclude signs and symptoms of relapsing forms of maxillary sinusitis and/or recurrence of OAF (Figure 3).

No intraoperative or immediate postoperative complications were observed. Among minor complications, nasal synechia was found in 3 patients (8.82%); recurrence was observed in 2 cases (5.88%), both suffering from diabetes mellitus (DM) and being tobacco smokers. The overall success rate after the primary intervention was 94.12%: no recurrent OAF was reported after the second attempt. Intraoperative cultures were performed in 27 cases (79.41%): positivity was found in 13 patients (38.23%) with a predominance of Gram-positive anaerobes. A specific antibiotic therapy guided by antibiograms was performed. A large fungal ball was removed in one case (2.94%) and odontogenic cysts in 6 cases (17.64%). Thirty patients (88.23%) completed the 12-month follow-up; all cases had a complete closure of the OAF, were symptom-free, and good ventilation in the maxillary sinus was found.

## DISCUSSION

The increased use of oral implants in the past three decades has led to an increase in paranasal sinus complications such as penetration/migration of dental implants and/or grafting materials into the maxillary sinus. Recent scientific evidence suggests that the increasing number of dental surgeries over the past few years may be associated with a raised incidence of iatrogenic sinusitis [18]. The infection is typically polymicrobial, with a large percentage of obligate anaerobes [19].

*Streptococcus pneumoniae*, *Moraxella catarrhalis*, and *Haemophilus influenzae* are the most common pathogens implicated in chronic sinusitis [20-23]. The most common symptoms and signs of sinus complications following dental surgery include facial pain, headache, nasal obstruction, swollen cheek, purulent rhinorrhea, post-nasal drip, cacosmia, hyposmia, paresthesia, and roncopathy [24-28].

OAF usually occurs when the Schneiderian membrane is interrupted by conditions such as the infection of the maxillary posterior teeth, maxillary dental trauma, pathologic lesions of the jaw and teeth, or by iatrogenic effects such as dental procedures (extractions or dental implant complications) and maxillofacial surgery procedures. Several authors showed that surgical procedures of the upper first and second molars teeth are the most frequent etiologic factor for OAF [29-32]. In our patients, the second molar tooth was the most involved and this is because the roots of the second molar are in closest proximity to the sinus floor [29].

For an accurate diagnosis of maxillary sinus disease, CT is the gold standard due to the high resolution and capacity to discern bone and soft tissue. CBCT is a relatively new tool that has become increasingly important in the diagnosis of sinus disease; it uses approximately 10% of the radiation dose of conventional CT but has a higher resolution compared to conventional thin-slice CT [11]. In our patients, we used both methods with a preference for traditional CT because of the higher cost of CBCT.

As already reported by Felisati et al. [33], the surgical management of odontogenic sinusitis, unlike other forms of maxillary sinusitis, requires a combination of intraoral and endoscopic approaches. Indeed, surgical success largely depends on primary closure of the defect and simultaneous recovery of normal sinus function through spontaneous drainage from the natural ostium. To date, buccal and palatal flaps are the most common solutions used for OAF closure [34-36]. OAFs <5 mm generally do not need surgery because of spontaneous closure [37], while a defect >5 mm in diameter can be surgically closed with buccal flaps. In our study, patients were treated with the buccal advancement flap procedure designed by Rehrmann, which involves the creation of a trapezoid mucoperiosteal flap and its suture over the defect.

The nasal endoscopic approach has several advantages to the previously used Caldwell-Luc technique [38,39]: it is a less invasive procedure and has the possibility of direct endoscopic control and treatment, thus allowing a surgical “toilette” and enlargement of the maxillary ostium to favor a rapid recovery of maxillary sinus functions that is the key for long-term success [40-42]. Furthermore, endoscopic approach allows to explore the other paranasal sinuses that may also be involved in the infective process.

In our series, all patients were treated with ESS combined with oral surgery in one-step by the same two surgeons (otolaryngology surgeon and oral specialist) and the incidence of complications was remarkably low [43-45].

Recurrence after combined surgical approach is possible. In our patients, recurrence occurred in two patients, both smokers and with a diagnosis of DM. As known, tobacco use has unfavorable implications in the postoperative period of oral and sinonasal surgery as it induces the release of catecholamines that favor peripheral vasoconstriction with tissue ischemia and delayed healing. Furthermore, smoking is believed to reduce the immune system response altering the activity of the neutrophils [46]. Similarly, patients with DM who undergo ESS and oral surgery have an increased risk for postoperative complications as DM favors greater susceptibility to infections, chronic inflammation, and less tissue tropism [47].

The main limitation of our study is the small size of our sample; larger studies are necessary to confirm our results.

## CONCLUSION

Our results confirm that simultaneous surgery with a combination of an intraoral and endoscopic approach can be considered the best strategy for the long-term restoration of normal sinonasal homeostasis in selected patients with chronic odontogenic sinusitis and OAF, guaranteeing an effective treatment with minimal complications in the short and long term.
